# Outbreak of NDM-5-producing *Klebsiella variicola* in intensive care units: an overlooked pathogen in Argentina?

**DOI:** 10.1128/spectrum.00672-25

**Published:** 2025-06-23

**Authors:** Francisco González-Espinosa, Diego Marcelo Maurizi, Dina Pedersen, Alejandra Soledad Oriani, Celeste Martinez, Jerson Andrés Martínez Lozano, Gabriel Gutkind, Marcela Radice, Daniela Cejas

**Affiliations:** 1 Universidad de Buenos Aires, Facultad de Farmacia y Bioquímica, Instituto de Investigaciones en Bacteriología y Virología Molecular (IBaViM), Ciudad Autónoma de Buenos Aires, Buenos Aires, Argentina; 2 CONICET (Consejo Nacional de Investigaciones Cientíﬁcas y Técnicas), Ciudad Autónoma de Buenos Aires, Buenos Aires, Argentina; 3 Hospital Municipal Dr. Leónidas Lucero, Bahía Blanca, Argentina; Universidad de Buenos Aires, Buenos Aires, Argentina

**Keywords:** *Klebsiella variicola*, outbreak, ST3029, ICU, Argentina, IncHI1B-FIB, NDM-5, DTR

## Abstract

Here, we characterized the first *Klebsiella variicola* isolates responsible for an outbreak in the intensive care units (ICUs) in a hospital in Argentina. Five isolates recovered from inpatients and sinks at the ICUs of one hospital in Bahia Blanca City were included. Identification and antimicrobial susceptibility tests were performed using an automated system (Phoenix). Besides, they were identified using two mass spectrometry systems (Bruker Daltonics and BioMérieux) and a multiplex-PCR for *Klebsiella pneumoniae* complex identification. Whole-genome sequencing (WGS) was conducted to confirm bacterial identification and to analyze their resistome and mobilome. Additionally, clonal and phylogenetic relationships were investigated among local and worldwide isolates. Based on the Phoenix system, all isolates were identified as *K. pneumoniae* and resistant to last-line antibiotics, consistent with a difficult-to-treat phenotype (DTR). All of them carried *bla*_NDM-5_. The isolates were identified as *K. variicola* by one of the Matrix-Assisted Laser Desorption/Ionization Time-of-Flight Mass Spectrometry (MALDI-TOF MS) systems and by multiplex-PCR. WGS confirmed all isolates as *K. variicola* subsp. *variicola* belonging to ST3029, an infrequent lineage among the genomes analyzed. They displayed fewer than six SNPs, which corresponded to a unique clone, and the phylogenetic analysis exhibited a close relationship with isolates recovered from an environmental source in Brazil.

**IMPORTANCE:**

*Klebsiella variicola* is an emerging pathogen in humans belonging to the *K. pneumoniae* complex. *K. variicola* identification by classical biochemical methods is challenging, and it is often misidentified as *K. pneumoniae. K. variicola* has been isolated from diverse clinical samples such as blood, respiratory, and urinary tract infections, and in different environments. The present work describes for the first time *K. variicola* isolates recovered in Argentina, which were resistant to the last line of antibiotics consistent with a difficult-to-treat phenotype and responsible for a hospital outbreak. In addition, the phylogenetic analysis exhibited a close relationship to environmental isolates from Brazil.

## OBSERVATION

*Klebsiella variicola* species belongs to the *K. pneumoniae* complex and presents two child taxa, *K. variicola* subsp. *variicola* and *K. variicola* subsp. *tropica* (https://lpsn.dsmz.de/species/klebsiella-variicola). Traditional identification methods, based on biochemical tests, misidentify *K. variicola* as *K. pneumoniae*; therefore, the clinical impact of this species as a human pathogen is underestimated (1). *K. variicola* bloodstream infections were reported for the first time in Mexico in 2004 as part of a pediatric outbreak (1, 2). *K. variicola* is intrinsically resistant to ampicillin due to the expression of a chromosomal β-lactamase LEN. Besides, multidrug-resistant isolates have been reported in clinical settings, producing extended-spectrum-β-lactamases and/or carbapenemases, such as New Delhi metallo-β-lactamases (NDM-1) (3, 4). Even if *K. variicola* has been described in South America, no previous reports are available from Argentina (5). This study aimed to characterize *K. variicola* isolates responsible for an outbreak in the intensive care units in a hospital in our country.

## MATERIALS AND METHODS

Five carbapenem-resistant isolates were recovered in November 2023 in one hospital from Bahia Blanca, Argentina (Table 1). Three isolates were responsible for bacteremia, two of them in pediatric patients admitted at the pediatric intensive care unit (pICU) and the third one in an adult patient admitted at the adult intensive care unit (aICU). pICU and aICU are located adjacent on the same floor of the hospital, sharing the nurse’s station and handwashing sinks. Out of the latter, two isolates were recovered from its sink trap and washbasin, respectively, looking for a common reservoir.

**TABLE 1:** Bacterial features and method accuracy for *Klebsiella variicola* identification^*a*^^,^^*b*^

Kv	Date / SI	Patient / age*	Antimicrobial susceptibility (MIC in mg/mL)												*K. variicola* identification (Y: yes/ N: no)					
			MEM	IMP	AZT	CAZ	CRO	AMS	CT	CZA	CIP	GEN	AMK	TMS	Phoenix 50		VITEK-MS	MALDI-TOF MS	Multiplex-PCR	WGS
I	11/09/23 pICU	F / 1	8	8	≤1	>16	>4	>16/8	>8/4	>8/4	1	>8	>32	>2/38	N		N	Y	Y	Y
II	11/13/23 pICU	M / 12	4	8	≤1	>16	>4	>16/8	>8/4	>8/4	1	>8	>32	>2/38						
III	11/11/23 aICU	F / 52	4	8	≤1	>16	>4	>16/8	>8/4	>8/4	1	>8	>32	>2/38						
IV	11/13/23 sink-trap (siphon)	–	4	8	≤1	>16	>4	>16/8	>8/4	>8/4	1	>8	>32	>2/38						
V	11/13/23 washbasin	–	4	8	≤1	>16	>4	>16/8	>8/4	>8/4	1	>8	>32	>2/38						

^*a*^ Kv: *Klebsiella variicola* isolate; SI: Site of isolation (pICU: pediatric intensive care unit; aICU: adult intensive care unit). F: female, M: male. *: years of age. MEM: meropenem, IMP: imipenem, AZT: aztreonam, CAZ: ceftazidime, CRO: ceftriaxone, AMS: ampicillin-sulbactam, CT: ceftolozane-tazobactam, CZA: ceftazidime-avibactam, CIP: ciprofloxacin, GEN: gentamicin, AMK: amikacin, TMS: trimethoprim-sulfamethoxazole.

^*b*^ –, indicates no patient data available, as these are environmental isolates recovered from hospital sinks.

Identification and antimicrobial susceptibility tests were performed using BD Phoenix M50. The isolates were subjected to identification using both MALDI-TOF MS MBT Compass 3.4 (Bruker Daltonics, Germany) and VITEK MS PRIME setup IVD (BioMérieux). The raw spectra identification was obtained using a Microflex LT mass spectrometer by flexControl-Microflex 3.4 software (Bruker Daltonics, Germany) and analyzed using *Klebsiella* MALDI TypeR web-app tool (https://maldityper.pasteur.fr/). In addition, the identification was confirmed by multiplex-PCR (6). The presence of *bla*_CARBAPENEMASE_ was investigated by multiplex-PCR (7).

Genomic DNA was extracted and subjected to whole-genome sequencing (WGS) using the Illumina MiSeq platform with a 2 × 151 bp paired-end approach. Reads were assembled using SPAdes, annotated using PROKKA, and analyzed using Kleborate and BLDB (https://github.com/klebgenomics/Kleborate; http://bldb.eu/). Plasmid replicons were detected using PlasmidFinder. To screen for the presence of IncHI1B-FIB-*bla*_NDM-5_-harboring plasmid, the reads were mapped against a local reference plasmid (pM40-NDM-5, Accession number PQ247031). Then, a comparison among assembled genomes and pM40-NDM-5 was constructed using Proksee (https://proksee.ca/) (8).

A single-nucleotide polymorphism (SNP) distance matrix containing the core genome of local isolates and a reference genome (Accession number ASM2052554v1) was obtained using Roary to investigate the clonal relationship among them.

A set of 1932 *K*. *variicola* subsp. *variicola* assembled genomes were downloaded from public databases (https://www.ncbi.nlm.nih.gov/datasets/genome/?taxon=244366, https://pathogen.watch/) and analyzed using Kleborate.

A core genome alignment was performed by Roary, including 182 *K*. *variicola* subsp. *variicola* randomly selected genomes in addition to all ST3029 isolates (n:10), with five from databases and five from local isolates. A maximum-likelihood phylogenetic tree was inferred with IQ-TREE following the GTR + F + ASC + G4 evolution model with 1,000 random bootstrap replicates. The tree and metadata were visualized using Microreact (https://microreact.org/).

## RESULTS

The isolates were identified as *K. pneumoniae* subsp. *pneumoniae* by Phoenix M50 and VITEK MS PRIME setup IVD. The other commercial system (MALDI-TOF MS) identified all of them as *K. variicola*. In this case, the analysis of raw mass spectra using *Klebsiella* MALDI TypeR supported *K. variicola*. Accordingly, multiplex-PCR for *K. pneumoniae* complex identification presented the expected *K. variicola* amplicon of 275 bp (6) . WGS analysis confirmed the identification as *K. variicola* subsp. *variicola*. The isolates belonged to ST3029 and presented less than six SNPs among them, corresponding to a unique clone.

**Fig 1:**
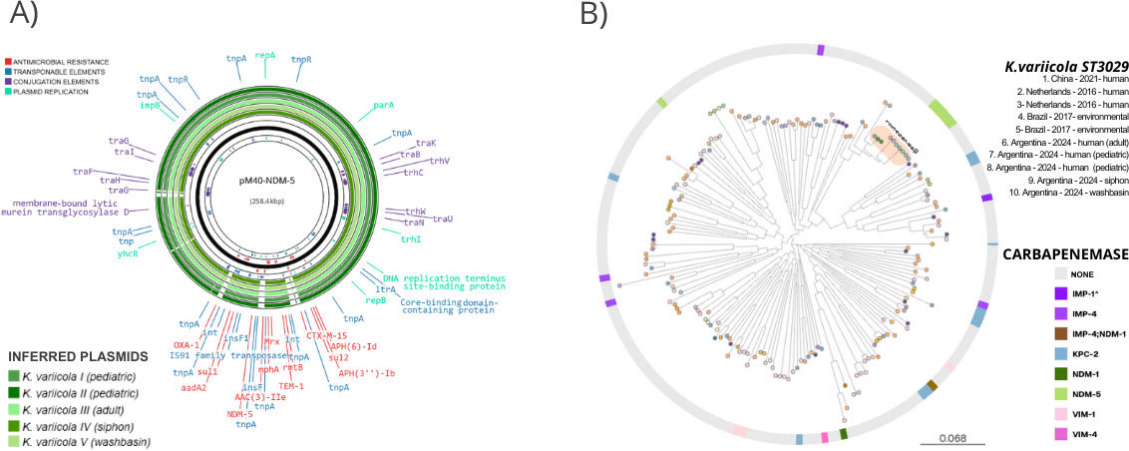
(A) Comparison among inferred *K. variicola bla_NDM-5_-*harboring-plasmids and pM40-NDM-5 (a local reference IncHI1B-FIB *bla_NDM-5_-*harboring-plasmid). Graphics were generated using Proksee. (**B**) Core genome phylogenetic tree of 192 genomes of *K. variicola* subsp. *variicola* (five from Argentina plus 187 worldwide). ST3029 isolates are highlighted using an orange oval. References in the external circle show the carbapenemase detected. This phylogenetic analysis can be visualized at https://microreact.org/project/kvariicola-subsp-variicola-argentina

The isolates were considered difficult-to-treat (DTR), displaying resistance to first-line agents, such as carbapenems, β-lactams+β-lactamase inhibitors, and ciprofloxacin. Also, they were resistant to trimethoprim-sulfamethoxazole, gentamicin, and amikacin (9). They were only susceptible to aztreonam (Table 1). All isolates harbored the same resistome consistent with the antibiotic-resistant profile, which included *bla*_LEN-16_, *bla*_NDM-5_, *aadA2, rmtB, strA, strB*, *qnrS1*, *sul1, sul2,* and *drfA12*. IncHI1B and FIB replicon plasmids were found. The presence of the *bla*_NDM-5_-harboring plasmid similar to the reference was inferred in all isolates (Fig. 1). However, to confirm the plasmid sequences and plasmid horizontal transfer capability, long-read sequencing and conjugation assays must be performed, respectively. The same capsular type (K61, *wzi3*20, and O-type O5) was predicted in the five isolates; however, no hypermucoidy markers, such as *rmpADC/rmpA2* nor other acquired virulence genes were detected.

Twenty percent (384/1932) of publicly *K. variicola* subsp. *variicola* genomes harbored an acquired *bla*_CARBAPENEMASE_, while *bla*_NDM_ alleles were detected in 100 isolates. In addition to five Argentinian isolates, another 22 carried *bla.*_NDM-5._ In our set of 1,932 genomes, 664 different STs were detected, with ST347 (n:119) being the most frequent, followed by ST3924 (n:45) and ST641 (n:41). However, *K. variicola* subsp. *variicola* ST3029 was infrequent, and only five isolates corresponded to it. Since 2016, this ST was detected in Brazil (n:2), Netherlands (n:2), and China (n:1). ST3029 isolates did not harbor any carbapenemase coding gene before, but two carried *bla*_CTX-M-14_. The five isolates from Argentina correspond to a new sublineage (SL) and a new cgST closest to cgST13941. A monophyletic group was formed between Argentinian and both ST3029 environmental isolates from Brazil; however, they belonged to SL1524. The remaining three ST3029 genomes were located together in an adjoining branch of the phylogenetic tree (Fig. 1).

## DISCUSSION

In 2004, *K. variicola* was proposed as a new species, representing about 8% of *Klebsiella* isolates*,* from both clinical and environmental samples (2). However, identification of the species within the *K. pneumoniae* complex, such as *K. variicola,* remains a challenge for clinical laboratories and requires updating the protocols to increase accuracy. In 2021, Barrios-Camacho et al. proposed a multiplex-PCR for the correct differentiation of the main bacterial species in the *K. pneumoniae* complex (6). In our experience, this multiplex-PCR was fully concordant with the WGS results. The overall performance of the Bruker MBT system provided higher accuracy for *K. variicola* identification, as previously reported. In Argentina, *K. variicola* identification is not routinely sought; therefore, we lack any clues for their dissemination as clinically relevant pathogens. Regardless of the sample origin (pediatric/adult inpatients or hospital environment), a clonal relationship was observed among all isolates included in this study. The present outbreak was duly contained by the replacement of the drainage pipes and by the disinfection of the washbasins by using bleach.

NDM-5 is one of the main carbapenemases reported in our region in addition to NDM-1, KPC-2, and to a lesser extent KPC-3 (10). *bla*_NDM-5_ was already reported within multi-replicons IncHI1B-FIB plasmids responsible for multidrug resistance dissemination in *K. pneumoniae* in Argentina (8). Accordingly, this genetic platform was inferred in the studied *K. variicola* isolates. To the best of our knowledge, there is no description in the literature of the ST3029, a lineage that had not been previously associated with carbapenemases, although two Dutch isolates carried *bla*_CTX-M-14_. Argentinian isolates clustered together with ST3029 susceptible isolates of environmental origin (*Acromyrmex* fungus garden) from Brazil. Hence, this species has been proposed to constitute a cross-kingdom bacterium, as it has been detected in a wide range of environments, revealing clear differences from other species of the *Klebsiella* genus, such as *K. pneumoniae* (11).

In conclusion, here, we describe for the first time *K. variicola* isolates in Argentina, which corresponded to a unique clone responsible for an outbreak in an adult and pediatric ICU. Besides, they corresponded to the first DTR ST3029 isolates reported worldwide.

*Klebsiella variicola* reads were deposited in the National Center for Biotechnology Information under BioProject accession number PRJNA1165358.
